# Improving the rehabilitation of individuals admitted to England’s National Spinal Injuries Centre with traumatic brain injury

**DOI:** 10.1038/s41394-024-00690-x

**Published:** 2024-12-18

**Authors:** Lawson Falshaw, Nigel King, Imogen Cotter

**Affiliations:** 1 The Oxford Institute of Clinical Psychology Training and Research, Oxford, England; 2 Buckinghamshire Healthcare NHS Trust, Buckinghamshire, England

**Keywords:** Health care, Neurophysiology, Neuroscience, Psychology

## Abstract

**Study design:**

Mixed methods service improvement project. Retrospective analysis of clinical documentation and qualitative focus group with clinicians.

**Objectives:**

Although traumatic brain injury (TBI) and spinal cord injury (SCI) often co-occur, many barriers have been found to identifying TBI in SCI rehabilitation and adapting treatment accordingly. This study aimed to compare the number of individuals with a TBI detected at England’s National Spinal Injuries Centre to figures found in previous research and understand the barriers to adapting SCI rehabilitation in the presence of TBI.

**Setting:**

England’s National Spinal Injuries Centre at Stoke Mandeville Hospital.

**Methods:**

This mixed methods study assessed the documentation at each stage of 88 patients’ treatment where a TBI could be detected and used to inform rehabilitation, and subsequently, a focus group was conducted with staff to explore the barriers to detecting TBI and adapting SCI rehabilitation.

**Results:**

Results suggested that data related to TBI were inconsistently recorded, the number of individuals recorded as having a TBI at the centre was lower than a recent study, and several barriers were interpreted from the focus group.

**Conclusions:**

TBI in SCI populations may be an invisible unmet need. Several barriers may exist which prevent clinicians from detecting TBI in this population and adapting rehabilitation accordingly. Findings have implications for rehabilitation for individuals with TBI and SCI admitted to the service.

Recent NHS data suggest that 50,000 people in the UK alone are living with a Spinal Cord Injury (SCI) [1]. These conditions can be life changing for the person directly involved, as well as having a ripple effect on loved ones around them, and wider economic impacts on society [2]. Although traditionally investigated separately [3, 4], one of the most commonly co-occurring conditions when someone suffers an SCI is Traumatic Brain Injury (TBI). Some research indicates that between 20% and 60% of people with a SCI will also have at least a mild TBI, particularly in those who acquire a traumatic SCI through motor collisions and falls [5–7].

Research suggests that having both an SCI and TBI poses unique rehabilitation challenges compared to having either condition alone. Firstly, individuals with both conditions may require additional adjustments during SCI rehabilitation, such as a longer stay in inpatient settings [8]. These individuals may also need early discharge planning and rehabilitation professionals and carers may need additional training to ensure more time and personalised support is provided so that the individual can safely return home [9]. Those with both conditions may also face the potential cognitive difficulties of a TBI. Processing speed, memory, problem solving and language skills may be impaired by a TBI, all of which are key in the learning involved in all aspects of the SCI rehabilitation process [10]. Depending on the severity of the injury, patients may suffer major cognitive impairments impacting their SCI rehabilitation. Some patients, however, may acquire milder cognitive impairments associated with mild TBI, and there is, therefore, a risk that these difficulties will be missed by rehabilitation professionals, thus limiting the therapeutic gains which can be made through rehabilitation [11]. Moreover, although most with a mild TBI will make a full recovery, a minority will still be recovering at three to six months, and a smaller minority will have long-term or permanent deficits which can impact on rehabilitation. Suffering a TBI in addition to a SCI may also have a greater emotional and relational impact on the survivor and their families as they adapt to life with both conditions [12].

The theory around ‘invisible disabilities’ after TBI underpins the current study, which suggests that individuals with TBI may not be perceived as disabled since their difficulties are not visually apparent. This invisibility can lead to misconceptions and unrecognised needs as external appearances do not reflect internal challenges. Thus, the theory would predict that clinicians in a spinal injury context may overlook the disabilities related to TBI due to their lack of visible cues, leading to potential under-detection and unmet needs for patients with both conditions [13].

Despite the high concurrence of SCI and TBI and the additional barriers faced by those with a dual diagnosis and their families, TBI in those with a spinal injury remains poorly detected. Previous literature has highlighted many barriers to spinal injury services in detecting and tailoring rehabilitation to patients with a TBI. For example, TBI often is not considered by rehabilitation professionals, is poorly documented, and cognitive screens may not be administered [14, 15]. One recent study by Sharma and colleagues [16] found that more than half of patients referred to inpatient settings for traumatic SCI rehabilitation had TBIs which had been missed, with more TBIs being missed in falls and assaults compared to motor accidents. Therefore, the researchers suggested rehabilitation professionals may have varying perceptions about how often TBI occurs based on the mechanism of injury. Nonetheless, recent guidelines state that SCI rehabilitation services should be screening for TBIs in this population; the clinical reference group, who set standards for the UK SCI rehabilitation centres has agreed, for the first time, for pre-screening of psychological needs associated with TBI to be part of their core recommendations.

## Service context

This project aims to assess and improve the detection and consideration of TBIs in those undergoing rehabilitation at the National Spinal Injuries Centre (NSIC) in Stoke Mandeville Hospital in Buckinghamshire, a 114 bed rehabilitation unit for people adjusting to life after a SCI. The National Spinal Injuries Centre (NSIC) at Stoke Mandeville Hospital has the clinical ability to admit new patients as soon as they are medically stable following an acute hospital admission. However, patients are often delayed because of bed capacity, which can be some months. Admissions typically range from three to seven months, depending on injury severity and rehabilitation needs. Discharge options include returning home with care support provided as needed. There can be delays in a care package being provided, in this case people are sometimes discharged to temporary (interim) nursing care. A minority of patients return to live permanently in a rehabilitation facility. A consultant led multidisciplinary team (MDT) meeting takes place weekly to discuss patient progress and coordinate care, focusing on holistic rehabilitation that includes physical recovery, psychological support, vocational training, and social reintegration. This meeting is informed by more in depth 4 weekly MDT wide goal planning meeting which are based on the Stoke Mandeville Spinal Needs Assessment Checklist and include the patient and a family member.

Although the NSIC is specialised in spinal injury rehabilitation rather than brain injury, due to the nature of SCIs, a recent study suggests many individuals admitted to NSIC with SCIs may also experience at least a mild-moderate TBI [17]. Furthermore, despite the clinical reference group’s new guidance, initial discussions with outreach and inpatient staff suggested TBIs are often not detected at NSIC or not recorded prior to referral, and are often not routinely screened for upon admission.

## The current study

Thus, this service improvement project aims to address the following questions emerging from the existing literature and the service needs at the NSIC: Firstly, is the number of patients reported to have TBI by the NSIC in line with national figures suggested by existing research? Next, what are the barriers to identifying and considering TBI during rehabilitation according to clinicians? The project subsequently aims to improve the NSIC’s detection and response to TBI in patients admitted for SCI rehabilitation.

## Method

### Phase one

The project was designed and divided into three phases based on consultation with senior NSIC clinicians and the involvement of an individual with lived experience of SCI rehabilitation at the NSIC. Phase one consisted of auditing the number of individuals with a TBI detected by the NSIC and comparing this figure with previous research.

### Participants

The records for 88 patients, newly admitted between June 2020 and July 2021 were screened. 65 patients were male (73.86%), 23 were female (26.14%), and patients’ ages ranged from 18 to 83 (*M* = 57.27 years, *SD* = 17.32). The most common type of SCI in the sample was traumatic SCI, affecting 51 patients (58%), and the most common mechanism of injury was falls, which was the case for 40 patients (45%).

### Design and materials

A quantitative, between-groups design was used to compare the number of individuals with a TBI recorded by the NSIC to a previous study. All documents were screened through electronic health records accessed through Trust computers. This study received ethical approval from Buckinghamshire Healthcare NHS Trust as a quality improvement project.

### Procedure

**Fig. 1:**
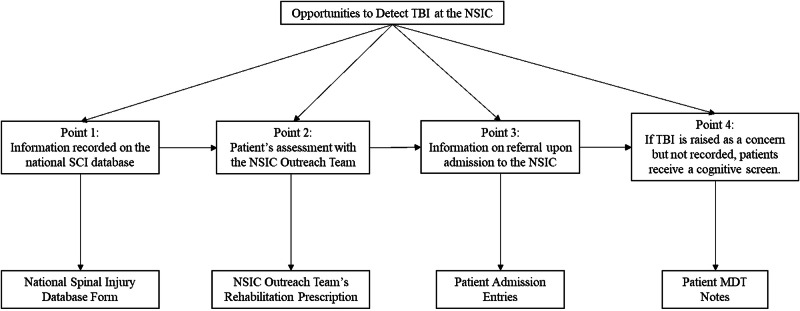
The different points at which TBI may be detected at the NSIC, and the corresponding documents screened.

The patient records were screened with reference to the four different opportunities that clinicians have to detect TBI throughout a patient’s journey at the NSIC. These stages are represented in Fig. 1 below. Firstly, the referral forms from the National Spinal Injury database were screened, as this is where all information related to spinal injury is recorded and then sent to the NSIC. Secondly, the rehabilitation prescriptions, records for each patient’s assessment by the NSIC outreach team were screened as these documents are passed on to the NSIC clinicians upon admission. Next, the admission entry for each patient was assessed, as this represents information about patients’ health at the time of entering the inpatient service. Rehabilitation prescriptions are written by senior members of the MDT following the outreach team’s assessments prior to an admission. Inpatient admissions assessments are conducted by the MDT within 24 to 48 h of admission. Finally, all of the weekly MDT notes for all 88 patients were screened. Additionally, the goal planning meeting documents for patients with a recorded TBI were reviewed, since if a TBI was identified at the NSIC, this should be discussed and recorded in these documents which involves considering patients’ physical health and psychological needs throughout their admission, and how these inform their rehabilitation. The Stoke Mandeville Spinal Needs Assessment Checklist forms the basis for goal planning and is a staff facilitated patient self-report measure that is completed within two weeks of admission.

The criteria for assessing whether a patient was deemed to have a TBI were pre-determined through the National Institute for Health and Care Excellence guidelines on the assessment of traumatic brain injury [18], and previous studies into TBI and SCI comorbidity [6]. A mild-moderate TBI was considered as a blow to the head resulting in post-traumatic amnesia (PTA) for under 24 h, a Glasgow Coma Scale (GCS) [19] score between 9 and 15, and/or loss of consciousness (LOC) of fewer than 15 min. A severe/very severe TBI was considered present if PTA was more than 24 h, a patient had a GCS score of below 9, there was LOC for more than 15 min, and/or there were positive results identified on a brain scan. Additionally, specific mention of TBI and/or cognitive difficulties was recorded.

### Analysis

The number of individuals with a TBI detected by the NSIC was compared with figures suggested by a recent study conducted at three spinal injury centres in Australia [17]. This was done by conducting a one sample proportions test in SPSS. The strategy used to search for papers to act as a comparator involved conducted preliminary searches on Google Scholar and consultation with experts in the field. Specifically, the expertise of the supervisors (NK, a Consultant Clinical Neuropsychologist and clinical lead of the local community acquired brain injury service, and IC, Principal Clinical Psychologist at the NSIC) were sought, as well as the Consultant Clinical Psychologist at the National Spinal Injuries Centre. This combined approach allowed the research team to gather relevant studies and expert opinions efficiently. The selected study, conducted by Craig and colleagues [17], aimed to determine rates of cognitive impairment in a sample of people with SCI with comparisons with individuals without SCI, and to determine how mood states vary from admission, through discharge, and after transitioning into the community in those with cognitive impairment compared with those with normal cognitive performance. This study was chosen as a comparator because it was the most recent large scale study exploring the number of individuals with a TBI in patients newly admitted for inpatient SCI rehabilitation across three SCI rehabilitation centres similar to England’s National Spinal Injuries Centre. It assessed patients throughout rehabilitation across three inpatient SCI rehabilitation centres in Sydney, Australia, over the 32-month period of the study between April 2010 and December 2012, and it assessed TBI comorbidity across all types of SCI which aligned with the present study.

### Results

The recent study selected as a comparator [17], aimed to determine rates of cognitive impairment in a sample of people with SCI, and to determine how mood states vary in those with cognitive impairment (including specifically cognitive impairment which could have been caused by a comorbid TBI) throughout their rehabilitation journey. They reported that 19 out of a sample of 88 patients with an SCI (21.5%) had a TBI. The results for phase one of the current study are shown in Tables 1 and 2. Significantly fewer individuals recorded as having a TBI were found compared to the previous study [17] (13.6%, *z* = −2.6, *p* = .009).

**Table 1:** Summary of indicators and documentation of traumatic brain injury (TBI) in the first 88 newly admitted patients at England’s National Spinal Injuries Centre from June 2020 to July 2021.

Criteria	Number of patients
Brain scan results indicative of TBI	0 patients
GCS scores	
* -* GCS 15	61 patients
- GCS 14	8 patients
- GCS 3	1 patient
- No recorded GCS scores	18 patients
PTA recorded	2 patients
Loss of consciousness upon injury	6 patients
Patients with recorded TBI diagnosis	11 patients
TBI recorded in goal planning meeting documents	0 patients

**Table 2:** Summary of cognitive impairments and neurological conditions recorded in the first 88 newly admitted patients at England’s National Spinal Injuries Centre from June 2020 to July 2021.

Category	Number of patients
Cognitive difficulties	28 patients
Confirmed TBI diagnoses among patients with cognitive difficulties	3 patients
Other conditions present in patients with cognitive difficulties	
Epilepsy	1 patient
Multiple sclerosis	1 patient
Long COVID and delirium	1 patient
Autism spectrum disorder (ASD), psychosis, obsessive-compulsive disorder, and history of substance misuse	1 patient
Guillain-barré syndrome	1 patient
Attention deficit hyperactivity disorder (ADHD)	1 patient
Encephalitis	1 patient
Historic hypoxic brain injury	1 patient
Patients referred for further dementia assessment	5 patients

### Phase two

Phase two is reported in line with the Consolidated Criteria for Reporting Qualitative Research (COREQ) Checklist. This phase involved conducting a focus group with NSIC clinicians to identify barriers to assessing for TBI and tailoring SCI rehabilitation accordingly, thus highlighting areas for service improvement.

### Participants

The sample was selected purposefully to ensure representation across the MDT. Contact was made with the lead of each discipline to identify outreach and inpatient clinicians from different disciplines and grades to be invited to volunteer for the focus group. A total of five out of nine invited clinicians attended the focus group, including one outreach clinician (a physiotherapist), and four inpatient clinicians, including one clinical psychologist, one medical doctor, one occupational therapist, and one physiotherapist. The facilitator (LF) was a male Trainee Clinical Psychologist conducting the research as part of a doctoral qualification in clinical psychology. The interviewer had no relationship with the team prior to the facilitator to reduce bias in clinicians’ responses during the focus group, although the interviewer spent time on introductions and outlining his interest in TBI and SCI at the beginning of the interview to build rapport with the participating clinicians.

### Design and materials

The structure of the focus group was informed by Krueger’s guidance on designing and conducting focus group interviews [20], as well as discussions with the supervisor who worked in the service (IC) and the individual with lived experience of SCI rehabilitation at the NSIC.

### Procedure

The clinicians met for 1 h via Microsoft Teams for the focus group, through which the meeting was recorded and transcribed. The interview questions were semi-structured and enquired about key factors clinicians would consider when assessing patients with and without a TBI, service expectations regarding TBI, how the NSIC’s processes meet the needs of people admitted with a SCI and TBI, the barriers to assessing and providing SCI rehabilitation for someone with a TBI, and what might overcome these barriers. No information was presented to the participating clinicians about the results of phase one. This was to avoid biasing responses, and because the focus group in phase two could identify a rationale for service improvement independent from the results of phase one.

### Analysis

The data from the focus group was analysed by (LF) in line with Braun and Clarke’s thematic analysis methodology [21]. This exploratory approach was chosen to allow the researchers to create meaning through NSIC clinicians’ comments, and to identify, analyse and report themes in the data to understand clinicians’ perspectives on TBI in SCI rehabilitation at the centre, and to understand the meanings of clinicians’ experiences within the wider social context of the service [21]. The data and analysis was also reviewed by the supervisors (NK and IC).

## Results

### Theme 1: barriers to identifying TBI in patients with SCI at the NSIC

#### Subtheme 1: systemic factors

Staff voiced pressures on the service which limit the extent to which clinicians can assess comorbidities: ‘*There’s something about …the system that we’re in and it being already under pressure and then, whether actually it’s fair for staff to flex their skills*.’’ Moreover, one clinician reported: ‘*With a TBI… I think on the stats that ends up looking like: ‘oh this patient’s stay’s been extended by two months’ and that’s seen as a bit of a failure rather than a really good example of how we’ve tailored our resources to fit that individual’s need*.’

#### Subtheme 2: brief referral information provided to outreach

Outreach staff mentioned the brief referral information they receive and the need to gather a lot of information: ‘*When we first get referrals, they’re usually ever so brief in every regard*.’

#### Subtheme 3: subjectivity

Clinicians also reported that rehabilitation in the context of TBI was subjective, reporting: ‘*I find it a wee bit subjective as to who with a cognitive impairment of a variety of diagnosis would and would not benefit’*, and ‘*I think that’s a wee bit subjective ‘cause what number on a MOCA can we meet?*’

#### Subtheme 4: delayed detection

Clinicians also noted that TBIs are not queried until later in rehabilitation: ‘*Patients in the adult population that have come with TBI as a diagnosis, it might be that it’s something becomes a bit more apparent later down the line during their rehab because it’s more mild*.’

#### Subtheme 5: lack of TBI understanding

Clinicians reported that they did not know how someone with a TBI attending the NSIC may present: ‘*The main areas which someone with TBI might be presenting with… I don’t know what all of them would be*.’

#### Subtheme 6: variety of TBI presentations

Additionally, clinicians spoke about brain injuries impacting patients in different ways: ‘*for someone with a TBI… each presentation is different*.’

### Theme 2: clinicians’ current rehabilitation for individuals with TBI and SCI

#### Subtheme 1: appropriateness for rehabilitation

Clinicians’ views conveyed a sense of confliction about providing rehabilitation for patients with TBI: ‘*They would not be appropriate to engage with intense rehab, because… there’s an element of whether they’ll be able to understand and follow the instructions, and how much we can offer, and how much they will gain from us*.’ Although equally, clinicians asked the question: ‘*If not us, then who?… is it better for people to get a little bit of what we have on offer even if it might not be the full package?’*

#### Subtheme 2: patients missing out

Clinicians voiced concerns about patients’ rehabilitation needs being met, stating: ‘*the other concern I have as well is the patient getting the right care and support and not missing out*’, and ‘*If you add in TBI… how you then cater that programme and have to backtrack to either give them more time or fit them in with how the rest of our population are working*.’ Additionally, they reported: ‘*Let’s go to neurological rehab’ may be the decision… I think that presumption there is that that service would be able to rehab their physical and their cognitive needs. I’m not overly aware as to how experienced in spinal cord injury rehab [neurorehabilitation] services are… but I hope that somebody can rehab them as a whole… ‘cause I’d hate them to be failed by one of two diagnoses*’

#### Subtheme 3: knts with TBI

Staff also reflected upon labels they may hold about patients with TBI and SCI: ‘*there is a risk for those with a mild TBI that they get early on labelled or experienced, as someone just unpleasant person or someone who just doesn’t listen.*’

#### Subtheme 5: the importance of goal planning

Clinicians noted the importance of the goal planning process: ‘*The goal planning process I think is very valuable… it might be that it’s hard to communicate how we set goals based on the TBI because that’s something we’re not familiar with*.’ Similarly, another clinician noted: ‘*With the goal planning… maybe it’s about that there should be some guidelines for people with complexities or multiple diagnoses and difficulties, including TBI*.’

#### Subtheme 6: limited opportunities for MDT working

However, staff also stated there were few chances to come together and discuss patients’ care: ‘*I think there’s a big value in the actual team working with that person to have a chance to talk together about that person, and I think there’s a bit of a gap in our system… it’s very rare you get much time where the professionals are there and the patient isn’t… you miss the opportunity for the MDT discussion*.’

#### Subtheme 7: impact of discharge process

NSIC staff also reported the additional challenges of discharge for patients admitted with TBI and SCI: ‘*For someone with a brain injury [discharge] can be very daunting and quite complex, and I’ve found that sometimes here we’re not overly good at communicating that, and I think having a brain injury on top can be can cause quite a lot of anxiety… So it isn’t just the day-to-day rehab, it’s as you approach discharge as well.*’

### Theme 3: improving SCI rehabilitation for TBI

#### Subtheme 1: need for TBI education

Clinicians reported that they would benefit from some education around TBI in SCI: ‘*I guess from an education point of view… the main areas which someone with TBI might be presenting with… I think knowing what to look out for to start with might be might be needed*.’ Another clinician agreed, mentioning: ‘*I think [TBI] is not as well understood. Certainly not for me, but, you know, across our whole team.*’

#### Subtheme 2: need for guidelines for rehabilitation

Clinicians were in agreement about the need for guidelines when working with someone with a suspected TBI: ‘*I think the most important thing is to have something in hand to recognise the signs pointing toward TBI.*’

#### Subtheme 3: importance of family

The clinicians also stated the impact of TBI and SCI on families and how the systems around the person can be a valuable source of information for TBI assessment and throughout rehabilitation: ‘*The guidelines should include something about how to engage family fully in someone’s rehab, but whether in the case of the query TBI, whether that might need to be particularly emphasised or more of an active decision amongst the team about who’s included in decision making at an early stage and why*.’

#### Subtheme 4: earlier adaptation of rehabilitation

Finally, the clinicians reported the importance of adapting rehabilitation earlier in a patient’s journey in the presence of a suspected TBI: ‘*Like [Psychologist] said to follow the guideline to if it’s expected… rather than waiting for the diagnosis. By that time, it may be really late… it’s better to have set guidelines and implement them before you have a specific diagnosis for that patient*.’

### Phase three

The final phase consisted of feeding back the findings of the audit and focus group to the service and giving recommendations for service improvement. The recommendations were decided upon based on gaps identified in the reporting of TBI-related information in the audit, and the themes raised by clinicians in the focus group. The service’s feedback on these recommendations was gathered, and lastly, a training package was developed for the NSIC based on the study findings and the service’s feedback on the recommendations.

### Feedback

The findings of the audit and focus group and subsequent recommendations were presented to the service at the NSIC’s monthly audit meeting in June 2022. A presentation was delivered by the research team to the service, which was transcribed and recorded. For phase one (audit), the key recommendations were: noting comorbidities on rehab prescription, completing all parts of admission entry, and expanding on TBI info in MDT notes, and adding TBI (or cognition) to goal planning sheet. For phase two (focus group), the recommendations were: a training package incorporating the perspective of someone with lived experience of rehab for TBI and SCI at the NSIC, a checklist to know what to look out for and what each professional should do, creating a space early on for professionals to discuss how TBI may impact goals, areas of risk, and discharge, and lastly, greater family involvement during assessment where TBI is queried.

The service’s feedback on the project and recommendations was positive: that the project had been helpful and will better equip clinicians to work with TBI. One piece of feedback was that there were concerns that TBI may be over diagnosed as a result of the findings of the project, and it would be important to upskill the clinical team on other factors which may result in the cognitive deficits which may lead clinicians to query TBI. Nevertheless, recognising any cognitive impairment early on, whether from TBI or other conditions, is important for the clinical team and could enhance the generalisability of the current study’s findings.

### Service improvement

Since education and understanding were key themes of potential improvement resulting from the focus group, a training package was delivered for the clinicians at the NSIC, which has been integrated into the NSIC staff induction. This was done in collaboration with a second individual with lived experience who was a nurse who previously attended the NSIC as a patient for rehabilitation for SCI in the context of a comorbid TBI. The training began with the individual with lived experience of SCI and TBI sharing their story and giving clinicians an exercise to hypothesise the resulting difficulties. Subsequently, the training covered the following topics: firstly, the ways in which TBI in addition to an SCI may impact an individual and their family, secondly, what to look out for, guidance around how TBI may impact the patient across healthcare disciplines, the individual with lived experiences’ feedback of care and how it could be improved. Additionally, a TBI summary checklist was provided summarising these points, and finally, information was provided regarding other factors which may impact cognition, following the NSIC’s feedback on the project at the audit meeting.

The training was recorded and included a pre-training questionnaire and post-training questionnaire, measuring clinicians’ knowledge of and confidence working with TBI in SCI, as well as how useful the training had been. This has been handed over to the NSIC to allow the package to be evaluated once it has been implemented.

## Discussion

This project aimed to compare the incidence of TBI at the NSIC to figures found in previous research and to understand the barriers to identifying and considering TBI during rehabilitation at the NSIC. Finally, the project aimed to improve the NSIC’s detection and response to TBI in patients admitted for SCI rehabilitation.

The results from phase one of the project suggested that the proportion of TBIs in the NSIC sample was significantly lower than even the lowest proportions identified in previous research. Data required to inform a TBI assessment was not reliably recorded, although the TBIs detected tended to be recorded in the outreach team’s rehabilitation prescription and inpatient admission entries. However, the TBIs recorded were not reflected in the documents throughout rehabilitation. For example, no patients with a TBI had their TBI mentioned in their goal planning sheets. The relatively small number of individuals with a TBI detected at the NSIC may reflect previous research which found that TBI is often under detected in SCI services, often due to the diagnostic criteria of TBI not being recognised [22]. For example, a recent meta-analysis with 92,780 patients has suggested the prevalence of concomitant TBI in patients with SCI is 32.5% [23]. As well as potential difficulties in recognising, for example, LOC, and PTA, in the NSIC sample the information related to TBI was not consistently screened and recorded. Inconsistent screening and varying documentation of TBI diagnostic criteria has also been found in other research throughout patients’ SCI rehabilitation [14] and may limit clinicians’ ability to identify TBI and modify SCI rehabilitation accordingly.

The results from phase two suggested that there may be several factors involved in detecting TBI at the NSIC and tailoring patients’ treatment accordingly. These were interpreted as: service pressures, staff perceptions, lack of understanding of how TBI may impact SCI rehabilitation, few opportunities for MDT discussion, subjectivity and a variety of TBI presentations, and a need for guidelines. These barriers are supported by Sharma et al.’s research which also found that rehabilitation professionals’ perceptions about how SCI may result in TBI may influence the detection of TBI in SCI rehabilitation [16]. Similar themes related to the importance of staff in considering TBI have been reported by Sommer and Witkiewicz, who noted that staff need basic education about the impact of TBI on patients with SCI [24]. Similar to the NSIC clinicians’ views, they reported greater emphasis is needed on staff knowing and assessing the cognitive, social and behavioural impacts of having both conditions on patients’ ability to engage in rehabilitation offered by different professionals in spinal injury rehabilitation MDTs.

### Implications and recommendations

In terms of the theoretical framework of the study, the themes interpreted from the focus group such as ‘lack of TBI understanding’, ‘staff perceptions’, ‘delayed detection’, and ‘patients missing out’ support the theory of invisible disabilities. Since clinicians reported not understanding all of the invisible symptoms of TBI, this may support the prediction that clinicians’ external perceptions of patients may not have matched the internal experience of patients [13]. A low proportion of TBIs was detected, which could have been because their difficulties may not have been seen through the lens of TBI. Moreover, as the theory predicts, clinicians reported patients may be labelled as ‘someone unpleasant or who does not listen’. Therefore, this study’s findings support the predictions that theory of hidden disabilities may make about clinicians holding beliefs that patients’ disabilities may only be perceived visually. This may prevent patients from being perceived as potentially having a TBI and limiting the extent to which these patients’ needs may be fully met in rehabilitation.

Based on the findings of the present study, the NSIC has incorporated some screening of TBI as recommended by the clinical reference group, however, there is still opportunity for improvement in the implementation and rigour of these screening processes. It is recommended that the NSIC standardise the way that TBI is screened and documented. This includes adapting the documentation at each stage to incorporate TBI and encouraging clinicians to consistently report information which may be relevant to assessing TBI. This could be done by adding comorbidities to the rehabilitation prescription, by ensuring clinicians complete all areas of the admission entry, and by adding TBI and cognition to the goal planning sheet template.

Moreover, given the lack of understanding of TBI noted in the focus groups, education is recommended at a team level to increase clinicians’ knowledge of what TBI looks like in patients at the NSIC and to be equipped to address the additional challenges that TBI may pose for SCI rehabilitation. This may be achieved through training, such as the package created for the NSIC being integrated into the staff induction. This could be accompanied by a ‘checklist’ which was suggested by clinicians, which may support staff to more thoroughly assess for TBI symptoms. Next, given the lack of space for discussion solely between professionals, it is recommended to create a space early in the patient journey to discuss how TBI may impact rehabilitation, adjustments required, risk, and discharge. Finally, the clinicians underlined the importance of the family in working with TBI in SCI rehabilitation, therefore the family should be at the heart of rehabilitation early on in assessment and throughout treatment.

These recommendations will be implemented by the NSIC in several ways. A meeting was held with the service to deliver the recommendations and offer ideas for implementation, such as editing the service’s documentation to ensure that information related to TBI is recorded for each patient throughout their rehabilitation in the NSIC. The training package has been integrated into the staff induction. Finally, a report was written which was given to the service which outlined the findings and underlined resulting recommendations for the service.

### Strengths and limitations

One strength of this project is that it adopted a comprehensive and systematic approach to comparing the number of individuals with a TBI detected by the NSIC to previous research. This was achieved by assessing the records which reflect each stage at which a patient at the NSIC may be assessed for TBI. This was triangulated with the focus group, which was useful in exploring the views of various clinicians and identifying areas for improvement ‘on the ground’. It also resulted in key recommendations and training for the service, addressing a common comorbidity treated by the service.

Limitations of this study include that the identification of TBI relied on patient records, which may not accurately reflect the clinical activity at the NSIC. If patients were identified as having a TBI, this should be recorded in the MDT notes, and then discussed in goal planning meetings. Only the goal planning documentation for patients with a TBI recorded in their rehabilitation prescriptions, admission entries, or MDT notes were reviewed, which could mean that there may have been additional patients with a TBI only recorded in their goal planning documentation. Nevertheless, this is mitigated by the fact that goal planning at the NSIC is an ongoing process and any identification of TBI throughout someone’s rehabilitations should have been captured in the MDT notes which were reviewed for all weekly MDT meetings for all 88 patients.

Furthermore, since the 88 patients in the NSIC sample were admitted from June 2020 to July 2021, there could have been a confounding impact of the COVID-19 pandemic. The COVID-19 pandemic significantly impacted service delivery at the NSIC. Bed capacity was reduced to ensure social distancing and infection control. The service for newly injured patients operated on about 80% capacity rather than its usual 96% capacity, elective (secondary) admissions were impacted, leading to delayed admissions and prioritisation of urgent cases. The use of telehealth services increased for consultations and follow-ups, such as the outreach team’s assessments resulting in the rehabilitation prescriptions, and strict visitor restrictions were implemented to reduce infection risks. Staffing challenges arose due to outbursts of infection impacting staffing and service delivery. Moreover, since many people’s daily lives were changed, and people were instructed to stay at home this may have had a confounding effect on the patients’ circumstances of injury (e.g. road traffic accidents), and their subsequent rehabilitation. This may mean that the 88 patients selected were not representative of the usual patients admitted to the NSIC, which could have influenced the number of individuals with a recorded TBI.

Moreover, it is important to be tentative when drawing conclusions from the comparison between the results of phase one and Craig et al.’s research [17], given the contextual differences between the two studies. Whilst their sample was drawn from rehabilitation centres in Australia, ours took place in the UK and was influenced by the COVID-19 pandemic, which may have affected the types of spinal injuries observed. Differences in injury profiles, healthcare systems, methodologies, and sample demographics could all influence outcomes and limit the generalisability of direct comparisons. Despite these limitations, comparing the present findings with Craig et al.’s research provides valuable context for understanding broader trends and discrepancies in the number of TBIs recorded in SCI rehabilitation. Without such a comparison, it is difficult to assess how TBI recording might vary across settings, which is crucial for informing SCI clinicians globally. Whilst the comparison should be interpreted cautiously, it enriches this study by situating its findings within a larger body of research and encouraging further exploration of potential international differences in TBI recording in SCI rehabilitation.

Furthermore, although the focus group captured the views of clinicians from the outreach and inpatient teams, the small sample of five clinicians from the centre may not have represented all of the perspectives of the wider MDT, which may have affected the qualitative views which were analysed in thematic analysis, which methodologically involves subjective interpretation.

Lastly, since the project was aimed at supporting clinicians at the NSIC, this limits the generalisability of the findings to other spinal injury services. As a result, future research could compare the incidence of TBIs with a larger sample, without COVID-19 restrictions, and an explore a larger pool of clinicians’ views. This may be done at different centres so that potential conclusions may be drawn across spinal injury services, which may confirm the representativeness of the findings at the NSIC to other centres.

## Data Availability

Additional data are available from the corresponding author on reasonable request.
